# Acute necrotizing encephalopathy of childhood: a single-center experience

**DOI:** 10.3906/sag-2102-47

**Published:** 2021-04-30

**Authors:** Erhan AKSOY, Ülkü ÖZTOPRAK, Halil ÇELİK, Fatih Mehmet Akif ÖZDEMİR, Mehpare ÖZKAN, Hülya KAYALIOĞLU, Ayşegül DANIŞ, Özge KUCUR, Selman KESİCİ, Mutlu Uysal YAZICI, Ebru AZAPAĞASI, Yasemin TAŞCI YILDIZ, Nesrin CEYLAN, Saliha ŞENEL, Deniz YÜKSEL

**Affiliations:** 1 Department of Pediatric Neurology, Faculty of Medicine, Dr Sami Ulus Maternity Child Health and Diseases Training and Research Hospital, University of Health Sciences, Ankara Turkey; 2 Department of Pediatric Neurology, VM Medical Park Pendik Hospital, İstanbul Turkey; 3 Department of Pediatric Neurology, Faculty of Medicine, Muğla Sıtkı Koçman University, Muğla Turkey; 4 Department of Pediatric Neurology, Faculty of Medicine, Abant İzzet Baysal University, Bolu Turkey; 5 Department of Pediatric Intensive Care, Faculty of Medicine, Hacettepe University, Ankara Turkey; 6 Department of Pediatric Intensive Care, Faculty of Medicine, Dr Sami Ulus Maternity Child Health and Diseases Training and Research Hospital, University of Health Sciences, Ankara Turkey; 7 Department of Pediatric Radiology, Faculty of Medicine, Dr Sami Ulus Maternity Child Health and Diseases Training and Research Hospital, University of Health Sciences, Ankara Turkey; 8 Department of Pediatrics, Faculty of Medicine, Dr Sami Ulus Maternity Child Health and Diseases Training and Research Hospital, University of Health Sciences, Ankara Turkey

**Keywords:** Febrile illness, encephalopathy, multifocal brain lesions, seizure(s), childhood

## Abstract

**Background/aim:**

Acute necrotizing encephalopathy is a rare type of acute encephalopathy characterized by multi-ocal brain lesions and associated severe neurological findings and various organ dysfunctions may accompany it.

**Materials and Methods:**

Patients with acute necrotizing encephalopathy of childhood diagnosed by pediatric neurology and pediatric intensive care at Sami Ulus Maternity, Child Health and Diseases Training and Research Hospital between 2007 and 2020 were included in this study.

**Results:**

Nine patients (six females, three males) with a mean age of 4.05 ± 1.94 years (age range 1–6.5) were included in this study. The interval range between fever and encephalopathy in patients was 1–4 days. Influenza A (3H1N1, one untyped) was detected in four patients, influenza B in three patients, and no cause was found in two patients. Major clinical findings other than febrile encephalopathy in all patients were a hemodynamic shock in seven patients, seizures in six patients, vomiting in five patients, dystonia in three patients, and flaccid paralysis in the upper extremity in one patient. Despite all our treatment approaches, including plasmapheresis, moderate to severe neurological sequelae was observed in all of our patients, who survived even with significant radiological improvement. Three patients for whom we could not perform plasmapheresis died.

**Conclusion:**

Our study revealed that thalamic involvement increased as the interval shortened, and brainstem involvement increased in patients over four years of age. The presence of persistent vomiting accompanying encephalopathy during the parainfectious period and plasmapheresis treatment being a treatment option that could prevent mortality were cautionary for our study.

## 1. Introduction

Acute necrotizing encephalitis (ANE) is a rare but severe disease with neurological complications triggered by possible viral or bacterial infections, with multifocal brain lesions, especially in the bilateral thalamus. Mizuguchi et al. first described the form seen in childhood (acute necrotizing encephalitis of childhood; ANEC) in 1995 [1,2]. Although it has been reported with high rates from Japan so far, as a result of studies conducted worldwide, ANE has been observed to be not specific to a particular race [1–4].

ANE often begins with upper respiratory infections (URIs), less frequently gastroenteritis, or acute otitis media [2–5]. Mostly influenza A, H1N1 (pdm09) and influenza B, along with parainfluenza, mycoplasma, chickenpox, human herpesvirus 6 and herpesvirus 7 (HHV-6 and HHV-7), enterovirus, reovirus, rotavirus, herpes simplex, coxsackie, measles, Rubella viruses, and diphtheria-tetanus-pertussis (DPT) vaccine are held responsible for the etiology. Recently, a case of SARS-coV2-related (COVID-19) ANEC has been reported [6]. Apart from these factors, genetic susceptibility (HLA DRB/HLA DQB) and environmental factors are other factors responsible for possible pathogenesis [2,5,7,8].

Despite the increasing number of studies in recent years, etiology and pathogenesis remain uncertain. However, the most common and accepted view regarding pathogenesis is the cytokine storm resulting from excessive cytokine release [5,8–11]. Proinflammatory cytokines are mostly triggered by viral infections. However, after some bacterial infections causing necrotizing encephalopathy, pediatric patients’ immune functions are temporarily impaired; therefore, several opportunistic viruses may cross the blood-brain barrier and cause an infection. According to cytokine storm hypothesis, cerebral involvement occurs secondary to blood-brain barrier dysfunction, endothelial damage and increased permeability due to several cytokines released, especially interleukin-6 (IL-6) and tumor necrosis factor-alpha (TNF-α). Various organ dysfunctions related to hypercytokinemia, such as shock and disseminated intravascular coagulation (DIC), liver dysfunction and acute renal failure, can be added to the clinical manifestation [5,8]. Although ANE is mostly seen as sporadic and nonrecurrent, fewer recurrent and familial (ANE1) cases have also been described [7]. Ran-binding protein 2 (RanBP2) is an essential protein for the energy metabolism of neuronal cells. The heterozygous missense mutation in the gene encoding RanBP2 (the most common c.1880C > T: p.Thr585met) creates a predisposition to ANE1 by causing energy breakdown. ANE1 is a rapidly progressive encephalopathy with incomplete penetrating OD inheritance. There are also ANE1 cases without RanBP2 mutation. Therefore, ANE1 has a complex heterogeneity [2,7,12].

In light of this information, this study has been discussed to share our center’s experiences regarding the diagnosis and treatment characteristics, clinical and radiological prognosis of our cases due to the rareness of ANEC.

## 2. Material and methods

### 2.1. Patients

Patients with ANEC diagnosed by pediatric neurology and pediatric intensive care (PICU) at Sami Ulus Maternity, Child Health and Diseases Training and Research Hospital between 2007 and 2020 were included in this study. The patients’ presentation symptoms, history of infection and vaccination, clinical, laboratory, neuroradiological findings, treatment and prognosis were retrospectively analyzed from medical records. 

The patients were categorized according to the Neilson criteria. According to this categorization, cases with clinical presentation in the form of febrile acute onset encephalopathy, absence of pleocytosis and protein elevation in cerebrospinal fluid (CSF), variable high values in liver function tests (LFT), and characteristic symmetrical multifocal magnetic resonance imaging (MRI) lesions, including bilateral thalamus involvement, were considered ANE. Patients with recurrent, familial, and ANE clinical symptoms without thalamic involvement on MRI were accepted as ANE1, a subtype of ANE [7,13]. Acute alteration in consciousness (range: somnolence-coma) and/or behavioral (e.g., confusion or irritability) changes were considered encephalopathy. The variability of consciousness level manifested itself from confusion to coma and was evaluated according to the Glasgow Coma Scale (GCS). Electroencephalography (EEG) was used in cases of encephalopathy or concomitant seizures. Especially acute disseminated encephalomyelitis (ADEM), which is mostly confused with ANEC in the differential diagnosis, along with cerebellitis, aseptic meningitis, viral or immune encephalitis, mitochondrial disease and other metabolic causes, Reye’ syndrome, malignancy, primer angiitis of the central nervous system, and autoimmune diseases were excluded by standard diagnostic tests (brain/spinal MRI and/or CT, EEG), blood and CSF analyzes. 

Yamamoto et al.’s ANE severity score (ANE-SS) was used to evaluate the treatment efficiency and prognosis of the patients. Accordingly, the presence of shock findings was scored as 3 points, being over four years old as 2 points, the presence of lesions in the brain stem on brain MRI as 2 points, platelet count less than 100 thousand, and CSF protein higher than 60 mg/dL were scored as 1 point each. This scoring was graded as low risk between 0 and 1 points, medium risk between 2 and 4 points, high risk between 5 and 9 points [14]. In the treatment, while intravenous (IV) pulse steroid was initiated in all patients, intravenous immunoglobulin (IVIG), plasmapheresis (PLEX), or their combination was added to some. Acute treatment onset time and duration, initial and control neuroimaging findings, clinical and radiological responses to treatment were evaluated.

### 2.2. Neuroradiological evaluation

Radiological protocols were applied on Philips Achieva 1.5 T magnetic resonance imaging (MRI) and/or Siemens Somatom 16-slice computed tomography (CT) device. A pediatric radiologist interpreted brain/spinal MRI scans of the children. The presence of pretreatment thalamus involvement, location, and distribution of multifocal lesions, limitation of enhancement and diffusion, presence of hemorrhage and/or necrosis were compared with posttreatment findings. IBM SPSS (version 21.0, IBM Corp., Armonk, NY, USA) was used to perform data analysis. Data were summarized by number (percentage) and mean ± standard deviation (or median and range). The groups were compared with each other. p ≤ 0.05 value was considered significant.

## 3. Results

Nine patients [six females (66.7%), three males (33.3%)] were included in this study. The mean age was 4.05 ± 1.94 (age range 1–6.5) years, and six patients were over four years old at the time of admission. Demographic, clinical, laboratory, radiological and treatment of the patients are shown in Table 1. 

**Table 1:** Characteristic of study patients.

	Age/sex	Presenting symptom	Infectious agent	Intervala	Abnormallaboratory testsb,c	Abnormal signs of cerebro spinal fluid	Radiologic findings (MRI)	EEG	Treatment	Outcome	Follow-up (month)/Relaps (month)
1	3 years 1 month/F	Alteration in consciousnessFeverURIVomitingShock	Unknown	3	Hypertranaminase(AST: 139)(ALT: 98)	N	ThalamusBrainstemCerebellumBasal ganglia	Encephalopathymultifocal epileptic activity	IVMP (5), *PLEX (7)IVIg (5)	Motor impairment (moderate)	12/-
2	1 year/F	Alteration in consciousness Seizures FeverURIVomitingShock	Influenza A	2	Hypertranaminase(AST: 146)(ALT: 70)	Protein: 78.5 mg/dL	ThalamusBrainstemCerebellumBasal ganglia	Encephalopathytemporooccipital epileptic activity(left)	IVMP (5), *PLEX (5)IVIg (5)	Motor impairment(mild)	1/-
3	4 years 5 month /M	Alteration in consciousnessSeizureFeverURIShock	H1N1	3	Thrombocytopenia(PLT:49,000/)	ND	Basal gangliaBrainstemTemporal	Encephalopathymultifocal epileptic activity	IVMP (5), *PLEX (10)IVIg (5)	Motor impairment (moderate)	3/-
4	5 years 1 month/M	Alteration in consciousnessseizuresFeverURIMyalji	Influenza BMycoplasma pneumonia	1	Hypertranaminase(mild)(AST: 62)(ALT: 56)	2 leukocytes/mm3Protein: 71.7 mg/dL	ThalamusBrainstemTemporal	Encephalopathy	IVMP (5). *PLEX ( 5)IVIg (5)	Motor impairment (mild)	1/-
5	6 years 3 month/F	Alteration in consciousnessseizuresFever	H1N1Mycoplasma pneumonia	1	Hypertranaminase(mild)(AST: 54)(ALT: 41)	Protein: 142 mg/dL	ThalamusBrainstemCervical cord (C4-C6)	Encephalopathy	IVMP (5) *PLEX ( 7)IVIg (5)	Motor impairment (moderate)	3/-
6	4 years 7 month/M	Alteration in consciousnessstatus epilepicusFeverURI	H1N1	2	Normal	ND	ThalamusCerebellumBasal gangliaTemporal	Encephalopathy	IVMP (5), *IVIg (5)	Ex(3.d)	-/-
7	5 years 11 month/F	Alteration in consciousnessFeverURIVomitingShock	Unknown	1	Hypertranaminase(AST: 92)(ALT: 71)	Protein: 99 mg/dL	ThalamusCerebellumBasal gangliaTemporal	Encephalopathy	IVMP (5), *	Ex	12(up to second episode)
8	1 years 4 month/F	Alteration in consciousnessseizuresFeverURIVomitingShock	Influenza B	4	Hypertranaminase(AST: 123)(ALT: 66)	N	ThalamusCerebellumBrainstemBasal ganglia	Encephalopathy	IVMP (5), *IVIg (5)	Ex	12 (up to second episode)
9	6 years 5 month/F	Alteration in consciousnessstatus epilepticusFeverURIShock	Influenza B	1	HypertranaminaseThrombocytopenia(AST:140)(ALT: 73)(PLT: 95.000)	1 leukocytes/mm3Protein: 115.9 mg/dL	ThalamusCerebellumBrainstemTemporal	Encephalopathymultifocal epileptic activity	IVMP (5), *PLEX (5)	Mental motor impairment(severity)	36/-

M: male; F: female; URI: upper respiratory infection; EEG: electroencephalography; AST: aspartate aminotransferase; ALT: alanine aminotransferase; PLT: platelet; N: normal; ND: not documented; IVMP: intravenous methylprednisolone; IVIG : intravenous immune globulin; PLEX: plasmapheresis; MRI: magnetic resonance imaging.*: oral steroid maintenance therapy after IVMP treatment.ainterval (time between fever and encephalopathy); b other etiological possibilities ruled out (including infectious, metabolic, autoimmune, and immune encephalitis); cthe normal value for ALT 8 to 33 U/L, for ALT 29 to 33 IU/L, and PLT 150,000 to 450,000/mm3. Mild impairment: not affecting the quality of life; moderate or severe impairment: requiring special treatment or education.ANE cases (5–8): case 5: sibling death story (with similar clinical picture);

The mean interval time between fever and encephalopathy was 2 ± 1.94 (interval range 1–4) days. The cause of fever was URI in eight patients (88.8%) and both URI and gastroenteritis in one patient (11.1%) during the parainfectious period. Using nasopharyngeal swab reverse transcriptase polymerase chain reaction (RT-PCR) method, influenza A (3H1N1, one untyped) was detected in four patients (44.4%), influenza B in three patients (33.3%), and no cause was determined in two patients (22.2%). In two cases with influenza B and H1N1 (cases 4 and 5, respectively), mycoplasma IgM serology positivity was also detected in serum. However, no other agents were detected in blood culture, blood serology, blood and CSF viral PCR tests in any of the patients. 

Except for febrile encephalopathy found in all patients, the main clinical findings were seizures (two status epilepticus, three generalized, one focal) in six patients (66.6%), and vomiting in five (55.5%). In addition to upper motor neuron examination findings in all patients, dystonia was present in three patients (33.3%) and bilateral flaccid paralysis in the upper extremity in one patient (11.1%). Signs of hemodynamic shock developed in seven patients (77.7%) at baseline or during clinical follow-up.

**Figure 1:**
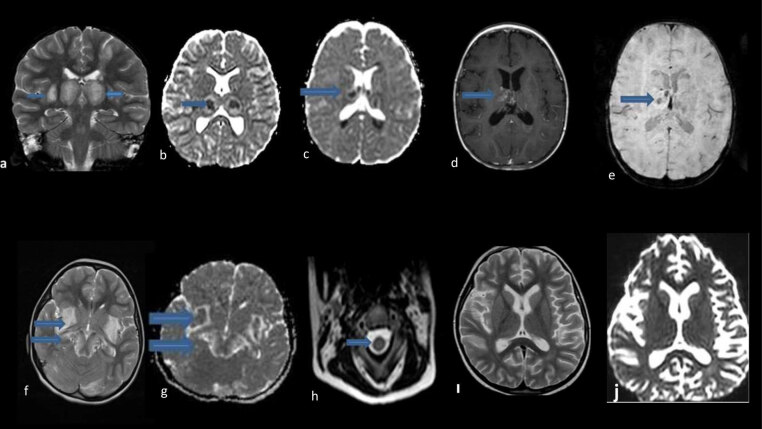
MRI images of our cases. a: in the coronal T2-weighted image of case 1, swelling and hyperintensity in the bilateral thalamic and external capsules and hyperintensity in the left parietal lobe; b: bilateral restriction is seen in the apparent diffusion coefficient (ADC) of the same patient; c: in the ADC of case 2 bilateral thalami and periventricular white matter restriction; d–e: postcontrast T1-weighted images of the same patient reveal more pronounced contrast enhancement pattern in the right thalamus and Hemosiderin deposition is observed in the left thalamus in susceptibility weighted imaging (SWI); f–j: case 5. (f) axial T2-weighted images demonstrating bilateral brainstem, medial temporal lobe, external capsule, occipital involvement, (g) markedly restriction in lesions described in ADC, (h) axial T2-weighted cervical images shows C4-C6 hyperintensity, (ı–j) axial T2-weighted images demonstrating and the apparent diffusion coefficient (ADC) control MRI lesions were seen to regress almost completely after treatment.

MRI findings are shown in Table 1 and illustrative examples in (Figures 1a–1j). The first brain MRI was performed on the first day of admission, and patients had a follow-up MRI following treatment after an average of 12 weeks (range 2–48 weeks). According to the MRI findings at the admission, bilateral thalamus involvement was found in eight patients (88.8%), brainstem in seven patients (77.7%), basal ganglia in six patients (66.6%), cerebellar in six patients (66.6%), and temporal involvement in five patients. While signal changes were less common in the hippocampus, internal capsule, external capsule, parietal, occipital, frontal, and white matter, cervical spinal cord involvement was observed in one patient (11.1%) (Figure 1h). Hemorrhage and/or necrosis were detected in five patients (55.5%), diffusion restriction in eight patients (88.8%) and enhancement in three patients (33.3%). Interval time between fever and encephalopathy was shorter in patients with thalamus lesions (p: 0.017). Besides, increased brain stem involvement was observed in patients older than four years old (p: 0.03).

The mean CSF protein of the patients was 84.7 ± 38.31 (29–142). In five (55.5%) patients, CSF protein level was above 60 mg/dL. No pleocytosis was found in the CSF in any of the patients. While hypertransanemia (from mild to markedly) was detected in seven patients (77.7%), the platelet value of two (22.2%) patients was below 100,000 (Table 1). The RanBP2 mutation could be studied in four patients, and no mutations were detected in any of them.

The patients’ median length of stay was three (range 0.5–28) weeks. Six of them (66.6%) were monitored as intubated on mechanical ventilation. In evaluating the severity of the disease, the average ANE-SS was 5.66 ± 1.94 (3–9), seven of them (77.8%) were in the high-risk group, and two (22.2%) were in the medium-risk group. The median immunotherapy initiation time was 1 (range: 1–5) days.

Five (55.5%) patients received IV pulse steroid, IVIG and PLEX treatment, two (22.2%) patients received IV pulse steroid and IVIG treatment, one (11.1%) patient received IV pulse steroid and PLEX treatment, and one (11.1%) patient received only IV pulse steroid treatment. When there was no clinical improvement in cases 3 and 5, PLEX was added to their treatment later. Simultaneously with immunotherapy, IV antibiotics, IV acyclovir, oral antiviral, and antiepileptic drug (AED) treatments for seizures were administered. A total of three patients in the ANE1 group died, with case no six on the 3rd day of the treatment, and cases 7 and 8 during their second attack occurred one year after the first attack. It was observed that PLEX treatment could not be applied to these three patients due to technical reasons. After acute attack treatment, all surviving patients were followed by a “slow” oral steroid taper over six weeks. The median duration of outpatient follow-up was 12 (range 0.5–144) weeks. According to the last examination, minor motor sequelae was found in two patients (22.2%) and moderate or severe motor sequelae in four patients (44.4%) (Table 1). 

## 4. Discussion 

ANEC is a critical and challenging pathology to manage in PICU. It is not uncommon, yet also underresearched. This study included nine patients with investigations for infectious sources, a clinical course based on laboratory and radiographic examinations, and a description of follow-up morbidity and mortality. Our findings showed that the majority of the patients had an identifiable viral infection, elevated liver function tests, and thalamic abnormalities, with high morbidity and even mortality if not treated by PLEX.

**Figure 2:**
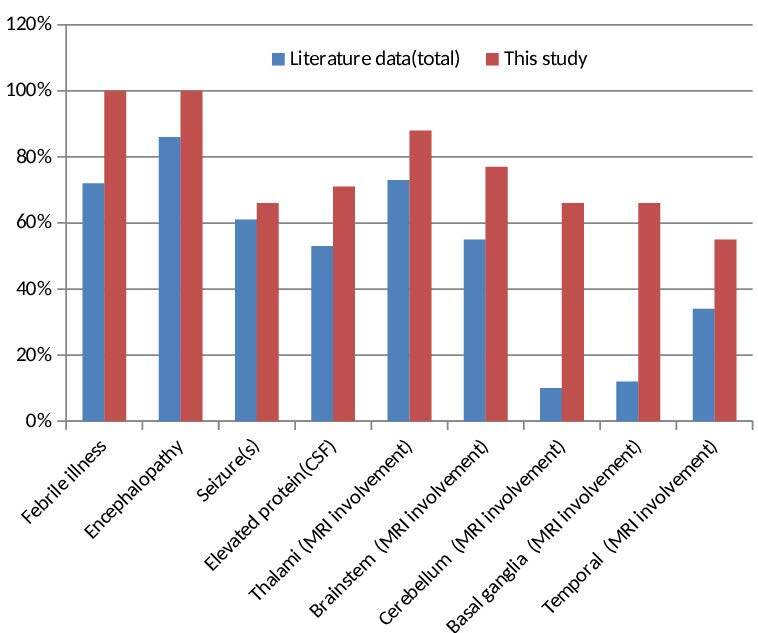
Clinical and imaging findings literature comparison.

ANE characterized by acute encephalopathy and multifocal brain lesions is a severe clinical presence [5,7,8,14]. One study reported that ANEC was seen in 4% of 983 children with acute encephalopathy [9]. Although etiology is not fully elucidated, viral or bacterial infections trigger hypercytokinaemia, which is responsible for the disease’s pathogenesis [2,13,15–18]. The prodromal phase has a heterogeneous clinic in the form of the encephalopathy recovery phase. The shortness of interval in the prodromal period causes the cytokine storm to progress rapidly and aggressively. Therefore, on the first day, deep coma, which usually requires mechanical ventilation, and then upper motor neuron findings develop promptly [2,13,16,19]. Frequently, seizures, vomiting, cough and organ dysfunctions accompany the clinical picture. However, these reported findings are not specific to ANE and are seen in many diseases [5,14,16]. Comparison of our patients with febrile illness, seizure (s), encephalopathy, abnormal CSF, and radiological findings with ANE and ANE1 data in the literature (n: 123, total % rates) summary reported on a case basis shown in Table 2 and Figure 2 [2,16,20–25]. 

**Table 2:** ANE/ANE1 with children cases (literature summary and our data).

Study	Clinical findings			CSF	Radiologic findings (MRI involvement region)				
Cases number (reference number)	Febrile illness	Encephalopathy	Seizure(s)	Elevated protein	Thalamus	Brainstem	Cerebellum	Basal ganglia	Temporal
Levine et al., 2020 (summary of cases 2003-2019 ) (2)	56	75	49	55	58	49	9	8	38
Marco et al., 2010 (19)	2	3	3	2	3	0	0	1	0
Britton et al., 2017 (20)	4	4	1	ND	2	0	0	1	0
Mastoyianni et al., 2006 (15)	3	3	1	1	1	1	0	0	0
Koh et al., 2019 (21)	3	3	2	2	3	3	1	1	0
Dadak et al., 2020 (22)	ND	1	1	ND	2	2	0	1	0
Lim et al., 2016 (23)	7	4	5	ND	7	1	ND	ND	ND
Kim et al., 2004 (24)	13	13	13	5	14	12	2	3	4
Total of all cases (123)	88	106	75	65	90	68	12	15	42
Our study (9)	9	9	6	5	8	7	6	6	5

CSF: cerebrospinal fluid; ND: not documented; MRI: magnetic resonance imaging.

Radiological findings are essential in diagnosis due to the difficulties experienced in autopsy and its progressive deterioration. MRI is more sensitive than CT. Lesions mostly show a symmetrical multifocal distribution. T2 weighted and fluid-attenuated inversion recovery (flair) MRI mostly shows hyperintensity in the bilateral thalamus and brainstem. Bilateral thalamus involvement is one of the most defining features of this disease [5]. These lesions mostly show hypointensity and diffusion restriction in T1 weighted [26]. Thalamic involvement is also seen in Japanese encephalitis. However, symmetrical thalamic involvement and brain stem involvement rarely occur compared to ANE [25]. In brain MRI, we found that eight (88.8%) of our patients had bilateral thalamus involvement, seven (77.7%) had T2 and flair hyperintensity in the brainstem (T1 hypointensity in five and two, respectively), and eight patients (88, 8%) had multifocal involvement accompanied by restriction (Table 1). It is also stated that involvement is observed less frequently in the cervical spinal cord, cerebellum, medial temporal lobes (insular cortex) and other regions in the ANE. These radiological findings are consistent with autopsy findings [5,7,21]. As the disease progresses in ANE, the progression of radiological findings from edema to petechial hemorrhage and necrosis correlates with the severity of clinical findings [5,21]. Therefore, serial brain MRI is needed. A study conducted by Kubo et al. showed that lesions occurred in the brain before neurological symptoms were revealed using an advanced MRI device [10]. This condition is emphasized due to the cytokine storm that starts in the subclinical period, causing blood-brain barrier damage and an increase in vascular permeability. Also, MRI has an essential role in evaluating the effectiveness of treatment. The shrinkage of the lesions increases the survival rate as an indicator of treatment effectiveness. Results in ANE are generally not satisfactory and have a wide prognosis range from complete recovery (<10%) to persistent neurological deficits and death. The mortality rate is approximately 30% [2]. Being <1 year of age, the presence of coma findings accompanying brainstem lesions, hemorrhage and necrosis in MRI, various high levels in LFT and high protein values without pleocytosis in CSF examination, and thrombocytopenia are among the poor prognosis criteria [2,3,5,7,12,14]. In the study of Yamamoto et al. conducted with 18 patients, they showed that 66.7% of the patients were in the medium-risk group, and they had a high risk of mortality or severe neurological sequelae. Observing the recurrence increased the risk of neurological sequelae [5,14]. It was shown that patients with normal CSF protein and LFT values recovered more quickly [16]. Compared with the literature summary data, while febrile illness, encephalopathy, seizure(s), CSF elevated protein values and thalamus, brainstem, and temporal lobe involvement rates were similar in our patients, basal ganglia and cerebellum involvement were observed to be higher in brain MRI (Figure 2). Also, hemorrhage and necrosis were detected in MRI findings in five (55.5%) patients, hypertransanemia in seven (77.7%) patients, and thrombocytopenia in two (22.2%) patients. While hypertransanemia was detected in 62% of the patients in a study, it was found at a rate close to our study [27]. Our study showed that two cases (cases 7 and 8) with recurrence died. At the same time, hemorrhage and necrosis were detected on brain MRIs in the frontal lobe of case 8 and in the thalamus of case 2. In the other three cases (1, 2, and 3) in which necrosis was detected in many brain regions, including the thalamus, it was observed that two of them had moderate and one had minor sequelae. As the ANE-SS values increased, it was seen that there was a trend towards moderate/severity sequelae or mortality. Besides, our case 5, who had a history of a sibling’s death due to similar clinical complaints, had common multifocal lesions, including very rare cervical spinal cord (C4-C6) involvement. It was observed that this patient had significant regression in control MRI lesions three months after IV pulse steroid + IVIG + PLEX treatments (Figures 1h–1j).

A quick evaluation of the anamnesis, examination, radiological, blood and CSF findings is critical in initiating immunotherapy as soon as possible, preferably within 24 h [17,19]. However, there is no consensus yet on standard treatment guidelines. It has been reported that acyclovir and appropriate antibiotic treatment, as well as early initiation of IV pulse steroid treatment as immunotherapy and administration of high-dose oseltamivir with steroid treatment, are most useful in intensive care conditions [28]. In addition to these, combined IVIG and PLEX treatment is recommended in selected cases [15,16,19]. Therefore, we applied PLEX treatment in six (66.6%) patients with severe clinical findings and diffuse radiological involvement in brain MRI (five combined with IV pulse steroid therapy and IVIG, one combined with IV pulse steroid). When there was no improvement in clinical findings in cases 3 and 5, it was observed that they had moderate sequelae in their follow-up, although PLEX was added to their treatment later. As mentioned before, excessive cytokine release, especially IL-6 and TNF-α, occurs in ANEC, just as in COVID-19 infection. We could not administer the anti-IL6 or anti-TNF α preparations currently available in our country due to the COVID-19 pandemic, considering the urgency of the treatment, since all of our cases were before the pandemic and the supply of these preparations required a prolonged effort at that time. However, instead of these preparations, we have been successfully applying PLEX treatment for the last 7–8 years to remove IL-6, TNF-α, and other proinflammatory cytokines from the circulation. As mentioned, our three (6–8) cases with ANE1 treated in previous periods when we could not technically apply PLEX died. This situation suggests that PLEX treatment, together with immunotherapy in the cytokine storm, is a very beneficial method for survival. Because our other case with ANE1 (case 5) has been followed up in an outpatient setting for 1.5 years, with significant resolution in follow-up MRIs and moderate motor impairment findings with PLEX applied differently from the treatment of these patients (Figures 1g and 1h). No RanBP2 mutation was detected in any of our ANE1 cases we could examine. Considering our patients who died, it was observed that ANE1 had a worse prognosis than sporadic ANE.

This study has several limitations. Concerning the number of patients, a large enough sample group was not obtained. Since this is a retrospective study, the statistical data’s homogenization has been difficult because of some deficiencies. Also, the follow-up periods were not long enough, and the follow-ups irregularly created a handicap in evaluating the process from diagnosis to prognosis. However, the cohort analysis reflecting the disease’s clinical importance, diagnostic methods, and treatment strategies, especially PLEX efficiency, suggests that this study has a strong aspect. There are still few reports of in-depth analysis in the literature. Therefore, although our study’s sample size was limited, there was still certain significance. 

In conclusion, it is not possible to conduct randomized controlled clinical studies because of its rare occurrence. The data obtained so far are based on the clinical experience of different centers. Therefore, it is necessary to increase the centers’ experience to ensure early diagnosis and treatment standardization and predict the prognosis. For this purpose, we think that our experiences will shed light on guiding other centers. In this context, according to our results, it was observed that thalamic involvement increased as the interval between fever and encephalopathy became shorter, and brainstem involvement increased in patients over four years of age. Besides, we think that resistant vomiting accompanying encephalopathy in the parainfectious period is a stimulus for ANE’s clinical presentation. Our other results were that, except for three cases in whom PLEX could not be performed and died, all of our surviving patients had neurological sequelae ranging from moderate to severe, despite the apparent radiological improvement observed within an average of three months from all treatment approaches, including PLEX. These findings show the severity of mortality and morbidity risks of ANEC, and PLEX treatment is a treatment option that can prevent mortality. Therefore, long-term outpatient clinical and, when necessary, radiological follow-up of our patients is critical in prognosis. However, more studies related to ANE are needed in terms of increasing the experience.

## Financial disclosure

All authors do not have any financial or personal relationships with other persons or organizations that may inappropriately influence (bias) their work.

## Ethics approval

The study was first approved by the University of Health Sciences, Dr. Sami Ulus Maternity and Children Training and Research Hospital (EC number: E-20/10-003).
